# The care, stimulation and nutrition of children from 0-2 in Malawi—Perspectives from caregivers; "Who’s holding the baby?"

**DOI:** 10.1371/journal.pone.0199757

**Published:** 2018-06-27

**Authors:** Melissa Gladstone, John Phuka, Shirin Mirdamadi, Kate Chidzalo, Fatima Chitimbe, Marianne Koenraads, Kenneth Maleta

**Affiliations:** 1 Department of Women and Children’s Health, Institute of Translational Medicine, University of Liverpool, Alder Hey Children’s NHS Foundation Trust, Liverpool, United Kingdom; 2 Department of Public Health, School of Public Health and Family Medicine University of Malawi, Blantyre, Malawi; University of Washington, UNITED STATES

## Abstract

**Introduction:**

Universal access to quality early childhood development and care is a fundamental part of Sustainable Development Goal 4.2.1. Research from diverse settings, including that in low and middle income countries, now demonstrates the positive impact that interventions to promote play, stimulation, early communication and responsiveness can have, not just on child development, but on long term gains in education and economic growth. International agencies have recently produced the Nurturing Care Framework for Early Child Development in order to promote this and to encourage countries to move the focus from child survival to child thrival. Research on the best methods of integration of these programmes into present care practices, particularly in African settings is still very limited.

**Methods:**

We used qualitative methods to gain an understanding of care practices (play, developmental stimulation, early communication and responsive feeding) for children 0–2 years. We conducted 18 focus groups (FGDs), six PFGDs (Participatory Research focus groups), 18 in depth interviews (IDIs) and 20 observations with caregivers in rural and urban settings of Malawi. We used a topic guide, audio-recorded the FGDs and IDIS and transcribed them in Chichewa or Yao and then into English. We coded data using an inductive approach to thematic analysis. We placed the data within a framework with the emerging major and minor themes. We conducted quality assurance for translations and coding frameworks through cross comparison of data and used respondent validation to check our results.

**Results:**

Malawian caregivers see children’s play as a consequence of good health and wellbeing, less an interaction that a parent undertakes to promote wellbeing and learning. Non-verbal communication and responsiveness with infants is clearly present and caregivers have many one-on-one interactions with young infants. Furthermore, many caregivers have good knowledge of feeding recommendations but cannot always follow these due to constraints on money. When children become mobile (toddlers), play is an activity done more often between children or with other caregivers such as siblings or grandparents.

Community members consider that caring for children from 0–2 years is a woman’s domain. Despite this, both men and women acknowledge the importance of maternal wellbeing in enabling good care of children. The present socioeconomic situation of most families in our study means that income generation and food security come first. Many mothers spend most of their time managing the responsibilities of daily living and have limited time to dedicate to children’s play, responsiveness and communication with their children.

**Discussion:**

Programmes promoted as part of the Nurturing Care Framework which provide advice on developmental stimulation, play, early communication and responsive feeding should ensure that topics within these programmes are culturally appropriate for the setting. Furthermore, programmes must not be an added burden to parents but be supportive to parents managing many responsibilities of daily living. Multi-sectorial approaches where both men and women are provided with knowledge but are also supported through programmes which address family finances, safe spaces for children whilst families are working, and family mental health and relationships may enable programmes to work more effectively.

## Introduction

It is estimated that over 250 million children are not reaching their developmental potential worldwide with most of these children coming from low-income settings where they are much more likely to be affected by multiple insults such as malnutrition, poverty, frequent and chronic infections and lack of stimulation [1]

A number of studies in low-income settings have demonstrated the positive effects of programmes promoting cognitive stimulation, early communication, caregiver sensitivity and responsiveness on child development, maternal child interaction and child nutrition [2]. Many programmes encourage a “scaffolding” approach where the child is encouraged to consider new options to extend thinking through adult facilitation and joint attention [3–6]. This can be both for developmental stimulation and for responsive feeding and nutrition [7, 8]. Most approaches that are studied promote training with parents through home visiting or in groups[6]. These are most effective when provided early on, and as regularly as possible [9–11]. UNICEF, WHO and the World Bank are promoting implementation of these programmes for children from 0–2 years through the health sector through home visiting. The new Nurturing Care Framework recommends the Care for Child Development module (CCD) as part of this approach [12, 13]. CCD includes simple illustrated recommendations to parents to (1) improve stimulation through encouraging play, (2) improve quality of caregiver-child interactions (responsive behaviours), (3) encourage language development (communication) and (4) responsive feeding (breastfeeding, complementary foods). The recent WHO UNICEF programmes proviso that the main carer may not always be the mother [14, 15] but promote a continuing relationship with one person “for whom the child is special”.

Very few of the studies published so far concentrate in detail on whether these programmes fit within the cultural and socioeconomic settings of the implementing countries. We know from anthropological literature that the cultural context of caregiving varies considerably and may play an important part in both the goals expected by parents for their children and the methods used to attain those goals[16]. Some early literature demonstrates differences in caregiver to child eye contact[17]. Other studies have emphasised how promotion of development of the child may be within wider family groups in more socially distributed models particularly in African than would be the norm in many Western settings [18, 19]. Theories in developmental science have generally moved away from the idea that development only happens in fixed stages to a systems model where the development of the child is embedded in a set of social systems as described by Brofenbrenner in his ecological systems theory[20]. This work has influenced researchers examining the interplay of the wider family group [17, 21, 22], community and wider society [23] on child development within cultural contexts including those in Africa.

International organisations that are rolling out these programmes do not often make it clear as to how and whether they are taking into account these situational differences. It is likely that the social milieu, the specific cultural and social facilitators and barriers to care of young children, if taken into account when adapting programmes, are likely to make them more effective in varied cultural settings [24]. More recently, there have been calls to prioritise research looking at integrated implementation of early child development and maternal, newborn, child and adolescent health and nutritional platforms. In particular, formative research into the potential barriers to scale up of interventions is high up the list of priorities of proponents of early child development interventions in global settings [25, 26]. This study aims to provide information about these barriers by understanding the perceptions of parents and caregivers in relation to the barriers and facilitators for the care of children from 0–2 years in Malawi. This information will enable better structuring of programmes for providing advice relating to developmental stimulation, early communication, responsiveness and feeding practices in a Sub Saharan African context.

## Methods

### Qualitative methodologies used

As our aim was to understand perceptions, subjective experiences and meanings of care practices in Malawi, we undertook a multi layered mixed methods qualitative approach to gain a richer data set, improve trustworthiness and dependability of the data and to enable triangulation. Our epistemological position is one of interpretive subjectivism based on real world phenomena and an understanding that the world does not exist independently of our knowledge of it. Within this, we also come from a position of relativism–a view that reality is subjective and differs from person to person[27]. Using different data collection methods enabled us to understand whether data from different perspectives including that which is “said” and “perceived” (focus groups and interviews) fit a similar coding framework and reality to that which was “observed” (participant observations). We therefore aimed to explore perceptions, experiences, understandings and observed reality of participants within different contexts to provide us with a wider perspective on the situation **[28]** which we did not feel we could specify in advance. Table 1 outlines these different methodologies used as well as the numbers sampled. We conducted 18 focus group discussions (FGDs) with mothers, fathers, grandparents to generate open discussion on community views and norms and to understand and hear arguments as well as mutual views **[29]**. We also undertook 18 in depth interviews (IDIs) with a similar sampling framework. These were conducted in a narrative format and provided an opportunity to gain detailed individual perspectives and discussion of more personal issues **[30]**. We conducted six participatory research focus groups (PFGDs) for a wider discussion of the specific advice provided within the WHO/UNICEF Care for Child Development (CCD) programme. Within these sessions, we conducted the CCD training and recorded feedback and discussion of training materials. We conducted these groups firstly as they helped to shape what we needed in the interviews and focus groups. We then conducted interviews and focus groups concurrently. We conducted the observational work lastly as the interviews and focus groups did not provide enough real time evidence on what caregivers did with their children.

**Table 1:** Sampling matrix providing numbers of participants for all methods used in the study.

Setting	Data collection method	Mothers of children 0–2	Fathers of children 0–2	Grand-parents of children 0–2	Total (individuals)
Rural (Mangochi district)	FGDs (6/group)	3 (18)	3 (18)	3 (18)	9 (54)
	IDIs	3	3	3	9
	PFGDs (3-6/group)	3 (18)			3 (18)
	Observations	10			10
Urban (Blantyre district)	FGDs (6/group)	3 (18)	3 (18)	3 (18)	9 (54)
	IDIs	3	3	3	9
	PFGDs (3-6/group)	3 (18)			3 (18)
	Observations	10			10
Total participants		98	42	42	182

Key: FGD = Focus group discussion, IDI = In-depth interview

() = total number of participants in FGD or participatory focus groups

### Sampling and recruitment

We purposively sampled from different groups of individuals in diverse areas in Malawi to gain perspectives of those from different religions, income generating approaches, gender and age. During October 2012, health surveillance assistants conducted a census of all families with children under the age of two years in the areas studied. From this sample of 420 possible child-carer dyads, HSAs then randomly sampled (through use of computer-generated randomisation) participants for the FDGs, IDIs and participatory focus groups (PFGDs) from each of the areas as described below.

### Sampling of caregivers

We utilised a purposive sampling framework in that we were keen to gain views from different sexes, ages of participants, locations. We then sampled mothers, fathers and grandparents from three regions of Mangochi through HSAs. Areas included; Malombe (a fishing village), Katema (an agricultural village with a majority Christian population) and Nankhumba (a mixed fishing and agricultural) of Mangochi district which also vary in the prominent religious groups present within the community (Christian/Muslim). We also purposively sampled mothers, fathers and grandparents from Chilomoni (urban Blantyre) who varied in their distance from the road and how urbane/semi urbane they were (Table 1 sampling matrix). We undertook sensitisation and information sessions prior to sampling from any community with community leaders. We provided information leaflets in Chichewa to leaders and to Health Surveillance Assistants in the areas. These groups then provided leaflets to families of children of 0–2 prior to recruitment.

### Research team and data collection

We conducted focus groups (FGDs), in depth interviews (IDIs), observations and PAR groups between September 2012 and May 2013. KC and FC conducted all FGDs and IDIs in either Yao (Mangochi district) or Chichewa (Blantyre district) or in some cases in English with health workers who preferred to speak in English. KC and FC were both young and female and had both had over 2 years’ experience in qualitative research interviewing techniques in mental health. KC had a diploma level qualification and FC had a BSc. Both had been and were working as qualitative research associates in the area of maternal mental health and child protection. Neither had a relationship with either caregivers or professionals prior to study commencement and had no previous experience with the Care for Child Development training (CCD) or early child development research.

We created a topic guide for FGDs and IDIs that we piloted on four occasions prior to the start of the study to check that we were enabling the most pertinent information to be gathered (**S1 Text:** Topic guides for FGDs and IDIs CCD Malawi). FGDs took place in a central location in each village (school, home, church or health centre) and took approximately one and a half hours and IDIs took place in the caregiver’s home and took approximately forty minutes. An additional note-keeper and supportive research assistant was present from the local area to enable ease of transcribing and remembering who was who when speaking during the focus groups. These were all audio recorded, transcribed and translated. We compared transcriber quality of translation by comparing a handful of the same transcripts done twice by both transcribers to enable consistent translation. The team discussed any inconsistencies. We recorded observations as field notes and typed these up after each session. We used materials from the CCD package during the PFGD group sessions in order to provoke discussion and gain feedback about the materials. We audio recorded this feedback and discussions.

Observations: We used observational methods to generate a description of the daily life of parents and caregivers caring for their children under the age of 2 years in rural and urban settings in Malawi. Research assistants used a semi-structured interval-sampling approach to generate descriptions of interactions[31]. The research assistant recorded the time, context and participants present and then at one minute intervals (S2 Guide for structured observations), the key interactions between the child and caregivers at one minute intervals were written down followed by a written interpretation note every minute and then a review note every ten minutes[32].

### Analysis of data

We went through a series of steps in conducting a thematic content analysis[33, 34]. Our first step was to familiarise ourselves with the data by reading and reviewing the transcripts, taking notes and sharing ideas and views on the data. At this stage, we generated some ideas for codes. We continued with data collection until we felt we gained saturation of themes within the data and no new themes were emerging. We coded transcripts as we went along to enable us to see when we reached saturation. Data (written field notes and verbatim) from the observations was added to the database after the focus groups and interviews and the coded using the same coding framework. We conducted thematic content analysis by using the software package NVIVO 10. Data from interviews, focus groups and observations all fed into the thematic framework that we identified. This enabled us to triangulate the data with more certainty.

The next step was to create a codebook. To do this, members of the team coded a number of transcripts from the focus groups, and interviews separately to create a list of themes and sub-themes that were emerging from the data. We created the coding framework iteratively from the data to prevent selectivity in the use of data and aimed to allow semantics within the data to drive codes. We coded sections of text several times if there were a number of different semantics within the text and coded as many potential patterns and themes as possible at this stage. The codes were shared and were discussed and modified within the research team (JP, FC, KC) prior to a final decision about our coding framework and coding tree. Three coders (MG, SM and MK) coded all data once a final codebook had been created. We then crosschecked between coders to make sure we (MG, SM and MK) were coding similarly. We then searched for potential themes within our listed codes and sub-codes and collated the data within these identified themes. We used mind maps and lists to create piles of themes. We compared data within themes and across sources (rural/urban, female/male, grandparents/parents) at analysis. We compared data from IDIs and FGDs with observational data to identify areas of similarity in themes and areas where there was less cross-over. We then devised a set of candidate major and minor themes which we defined and refined once we placed within Bronfenbrenner’s framework.

**Fig 1:**
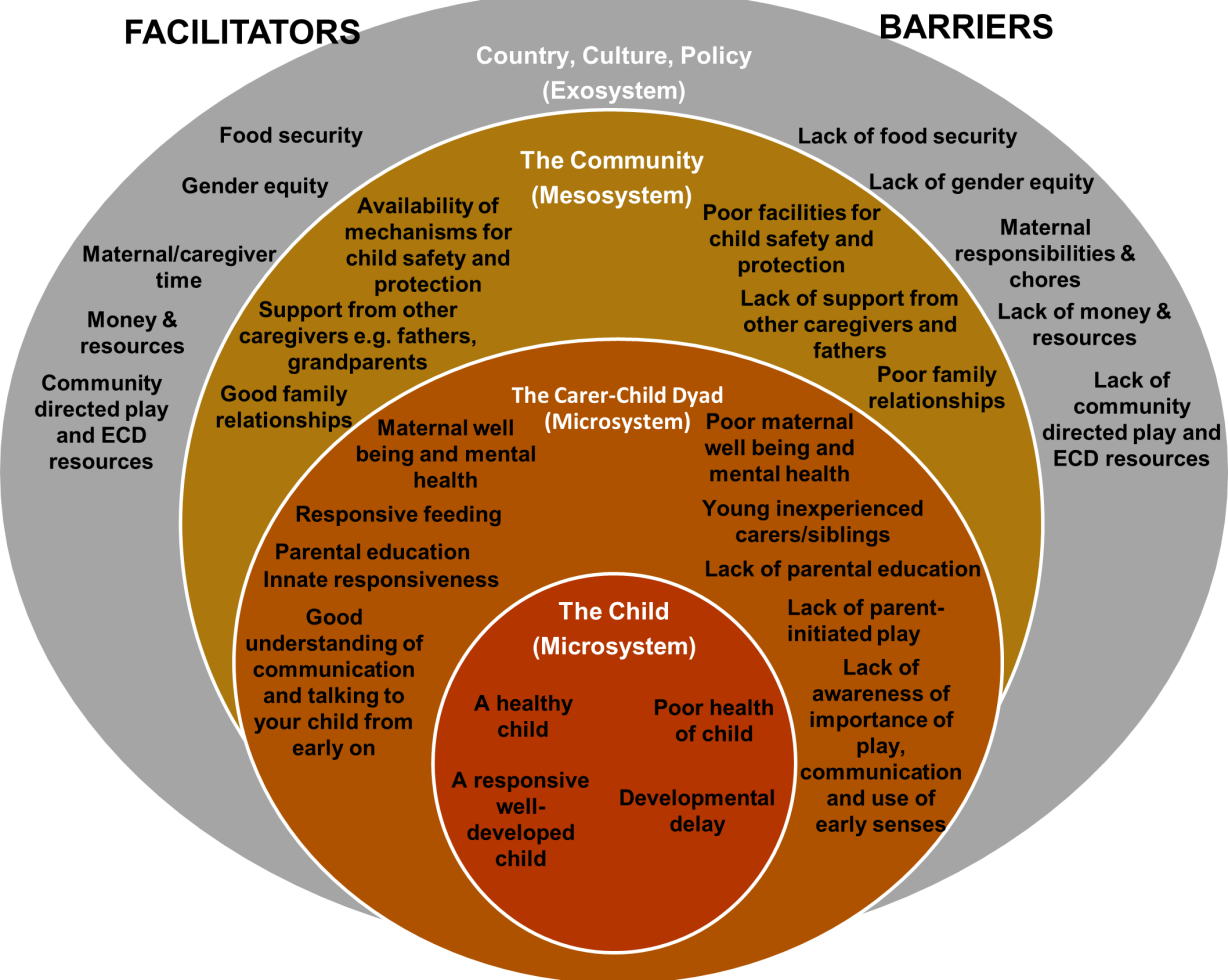
Facilitators and barriers relating to play, developmental stimulation, early communication and responsiveness within the ecological framework for children from 0–2 years in rural and urban contexts in Malawi.

We have utilised the Ecological Systems Theory conceptualised by Bronfenbrenner as a suitable framework through which to examine the themes which emerged from our data [35]. Bronfenbrenner’s theory or child development is commonly illustrated as a nested system of ‘environments’ presented as concentric circles (Fig 1) [36]. These interrelated systems include the ‘microsystem’–the interpersonal relationship and physical setting directly experienced by the child (family, peer group, school and neighbourhood), the ‘mesosystem’–connections between these, the ‘exosystem’–connections between settings that do not contain but may directly influence the child; and the ‘macrosystem’ which is described as the overarching pattern and fits all of the above [37].

For adaptation of the Care for Child Development package (see Table 2), we distilled the themes and subthemes from the framework in a way that could also be utilised for package development for our setting. An example of this might be the major themes of “child protection” and “maternal health and well-being”. After analysis of the data, we reviewed emerging themes and made consensus decisions about which of these could be best practically integrated into a 12 module-training package.

**Table 2:** Framework and major themes in the care, play and early communication of children less than 2 years in Malawi.

Ecological framework	Influence within framework	Major Themes adapted for the Care for Child Development Training Package
The Microsystem	The Child	Importance of health
		Purpose of play—A child who plays will be well
		Knowledge of infant hearing and vision
		Age and development of child
	The Carer Child Dyad	Parent initiated play
		The use of materials in play
		Maternal well-being and mental health
		What is responsiveness
		Communication and talking to your child from early on
		Responsive Feeding
The Mesosystem	Community care	Child protection and safety
		Who else looks after the baby
		Fathers roles
The Exosystem	Societal expectations	Maternal responsibilities
	Social welfare	Food security and resources

### Reflexivity

As a developmental paediatrician, MG is a strong supporter of interventions to improve early child development of children and was conscious of this bias during the study period. She is a British Caucasian researcher who is aware that her cultural bias may have affected the analysis of the research. JP and KM also both had strong positive views of interventions to improve early child development due to his previous studies and work in public health in the field of early child development and nutrition. KC, FC, SM and MK had no experience of previous interventions and had important roles as neutral interviewers and members of the analysis team.

We have utilised the COREQ guidelines to enable us a framework to check our reporting of the methodology and results of this study [38] (S1 Table: Supplementary Table 1 ISSM_COREQ_Checklist).

We gained prior informed consent from the community leaders as well as written consent from each individual who took part prior to any data collection. We provided each individual with an information leaflet relating to the study, which we read out to him or her again prior to consent. They then signed (or fingerprint stamped with a witness) an individual informed consent form prior to being enrolled in the study. We did not give written transcripts back to the participants. We did gain some respondent feedback at the end of the study through a series of four feedback groups where we explained the emerging themes from our research. These sessions included professionals and caregivers in each location where the research took place. During these sessions we asked participants to provide their views, feelings and experiences in light of our results. We took notes on the comments received during the groups and reviewed our themes and sub-themes in light of these sessions. They also enabled us to feel more confident that our research results were credible with members of the community.

This study was approved by the University of Liverpool Research Ethics Committee (RETH000536) as well as the College of Medicine Research Ethics Committee in Malawi (P.03/12/1193).

## Results

### Demographic features of the population

The sampling matrix in Table 1 shows the range of mothers, fathers and grandparents from the different areas. Mothers varied in age from 18 to 42 years with the majority (62%) ranging from 21–30 years. They worked as housewives, farmers and women running small businesses. Fathers also ranged in age from 18 to 42 years but were slightly older in either the 21–30 year or the 31–40 year age bracket. Grandparents were all over the age of 45 years. Men worked solely as farmers in 15% of cases but also worked as businessmen, building foremen, carpenters, drivers, bicycle repairers, cleaners and watchmen.

We present the results of our thematic analysis in Fig 1, which provides a schematic representation of the facilitators and barriers to play, developmental stimulation, early communication and responsiveness within Bronfenbrenner’s ecological systems framework.

We also demonstrate in Table 2 the major themes identified in the research. We will outline these in more detail in the subsequent paragraphs. These themes were then extracted into headings for each group workshop for our adapted Care for Child Development training programme for Malawi. These themes are highlighted in Table 2.

### The microsystem

#### The child

Table 3 outlines the main barriers and enablers to care, responsiveness, play and stimulation at the level of the child with some examples from the data, which illustrate this. We then provide suggestions as to how future programmes can adapt the Care for Child Development package in order to address the emerging themes. Most of the major themes here are provided from interview and focus group data. Much of this was also implicit in the observations but has been harder to capture in the way of quotes. We outline our results with more detailed quotes in the subsequent paragraphs.

**Table 3:** Barriers and facilitators at the level of the child of 0–2 years that affect integration of the care for child development package in Malawi.

Themes within the child	Barriers(quotes) and Facilitators (quotes)	Derivation of data (FGD, IDI, Obs)	Implications for intervention development
**1.Importance of health**	“*First of all*, *for a child to play well we need to notice how it is*. *If a child wakes up well*, *it will play and if it didn’t wake up well*, *you will notice and it won’t play*. *It is then our responsibility to take it to the hospital so that a doctor helps it and is able to play*” (FGD mothers rural).	IDI, FGD, Obs	Promotion of play as an important indicator of whether the child is well.
**2.Play supports health of the child**	*“The importance of playing for children is that*, *they make their body stronger and they as well learn that; something sticks in their head that they are now developing the personality skills*”(FGD mothers rural).	IDI, FGD	Material to include promotion of play as promoting healthy children.
**3.Age and Mobility of the Child**	“*Its right at the beginning…if you throw it in the air without dropping it makes the child happy”* (FGD grannies urban) *“When they are a year old and 4 or 5 months*, *this is when it knows that its friends are running away from it and it should follow them*. *It knows that if I was crawling I must stop and begin to walk to be able to run after my friends (*FGD mothers urban*)*	IDI, FGD, Obs	Encouraging play and communication and talking to your child by caregivers even before the child can walk.
**4.Knowledge of hearing and vision**	*“When you drop a tin or something…and then it makes noise you will note that the child gets shocked by the noise and then you know that he has started hearing” (*Mother, Chilomoni).	IDI, FGD	Providing specific information on the promotion of the use of senses (hearing and vision) from early on by caregivers.

**1. Importance of Health in Enabling a Child to Play: “Play reflects that the child’s health is fine and that he is happy” (Grannies FGD rural).** Carers describe play as something that can only occur if a child’s basic needs are met. If the child is well, bathed, cared for and happy, then they will play; “*If a child has reached a stage of eating porridge then you make and give it to him/her*. *If it happens that you have bathed the child ……then a child plays since has had enough in amount*” (Mother’s FGD rural)

**2. Play supports health of the child.** Many carers described play as a reflection of a child who is healthy and well and who is growing properly. “*to me as a parent I think playing is important to a child because it strengthens the body even the blood flows properly*” (Mothers FGD Urban). The child’s willingness to play or lack thereof is a marker of health.

**3. Age and development/mobility of the child.** The age of the child affects what play might be. A younger child might be swung around and played with; “*When you are swinging the child you see that the child tries to touch your mouth with his/her hands like this*, *but when you play with his/her mouth the child smiles showing happiness” (Mothers FCD rural)*. Play is something that a child might do once they are mobile; something that a child goes out to do with others. Crawling is a step in the right direction to be able to play and walking enables a child to actively play; “If *the child is walking we just praise God for that and pray that he/she should not fall sick now and again so that he/she can go to play*” (FGD Fathers rural).

**4. Knowledge of infant hearing and vision.** A number of parents and carers did not know that children could see and hear from early on but many health workers also did not have this knowledge. Old beliefs may still exist in some places; *“Ancient parents …would put the child in an enclosed environment where only adults could go in*. *this means that the child was in silence*. *After 6 months*, *they would bring it out and take it to the maize mill*, *at the mill makes its sound*, *the child would cry* … *and they would conclude that the child can now hear*. *Or they would take it to drummers and as they beat their drums*, *they would conclude that the child’s ears are open*. *These are parents of old but we parents of today believe a child can hear from birth* (FGD Grandparents Urban).

#### The carer-child dyad

Table 4 describes the barriers and enablers to care, responsiveness, play and stimulation at the level of the parent-carer dyad and how changes to the package and implementation may enable better support in the future through the use of this package. Interviews, focus groups and observations all supported these same themes. These are then discussed in more detail in the subsequent paragraphs.

**Table 4:** Barriers and enablers to care, responsiveness, play and stimulation at the level of the parent-carer dyad.

	Barriers and facilitators (quotes)	Derivation of data (FGD, IDI, Obs)	Implications for intervention development
**1.Parent initiated play**	*“Playing with children means that*, *you take a child and throw him/her into the air and you will see that a child is smiling or not*. *(FGD Malombe)”* *“The way I play with this child is by singing then you will imitate—when we are talking he also imitates talking following what you are saying” (Mother*, *IDI Mangochi)*	FGD, IDI, Obs	Promotion of chatting and conversing with the child as much as possible from early and not only doing physical play with a baby.
**2.The use of materials in play**	*“These children play with sticks and carry them at their backs … we also mould clay they play with the moulded clay toys* ….” “*A mother cannot afford to buy those…*..*we are talking about poor people*” (FGD Mothers Mangochi). “*The mother is taking his made plastic ball and is giving it to Q*. *Q is taking the ball and is throwing it away*. *The mother is telling Q to go and get the ball but Q is still breastfeeding” (Obs Mangochi*)	FGD, IDI, Obs	Home-made toys and materials can be promoted for use; dolls, water, soil, clay, hose pipes, rattle made from bottle and stones, wire bicycles, cloths, miniature kitchen equipment–burner, pots, papers, wood etc.
**3.Maternal health**	“*Maybe those children are very young not so*, *sometimes people may give birth to children of the same size…which it is difficult to take care of these children at once*”. *FGD Mothers Mangochi)*”.	FGD	Integration with messages about family planning and child spacing (linking programmes with health)
**4.Responsiveness**	“*Sometimes your heart can tell you that the child that is crying is yours” (PC FGD Chilomoni)* *“The mother is looking at D’s face and D is smiling*. *They are both holding hands*. *The mother is making him laugh hence D is now laughing”*. *(Obs Chilomoni)*	FGD, IDI, Obs	Most parents have an innate understanding and responsiveness with their child.
**5.Communication, talking from early on and joint attention**	“*Since they are just babies*, *they can’t tell us that they have heard what we are speaking to them*. *When it cries we go ‘‘lululu”*, *we throw it in the air and then it gets silent*, *meaning that it has heard us*.” (FGD Grandmothers Chilomoni). “*The second thing is that*, *when you take the child…*.*the child looks at you and when she sees bright things*, *you will see that the child will be looking …and…*. *you know that the child is seeing…*. *as you always stay together…and… you walk together*” (Fathers FGD Malombe, Mangochi)	FGD, IDI, Obs	Talk to your baby from early. Babies can hear and see from early on and their brains will grow if we speak and interact with them. Responsivity, non-verbal communication is important. Supporting mothers and caregivers in talking to their children through providing time for the caregiver to do this.. Supporting first time mothers
**6. Responsive feeding**	“*Like exclusive breastfeeding*,*…*.*a mother has gone to the field and has left the child with a fellow child and you find that the time she was supposed to breastfeed the child she hasn’t because she was in the field*. *You find that the baby feeds on porridge and only once in a day when she is supposed to feed the child frequently” (IDI Mangochi)*. *“It takes much time when I am breastfeeding the child so that he/she should have enough in amount*. *The child stops by himself/herself after he/she has enough in amount*.*”(IDI Mangochi)*.	FGD, IDI, Obs	Promotion of nutritional supplementation programmes within the locality. Supporting mothers for first 6 months so mother has capacity to exclusively breast feed

**1. Parent Initiated Play.** Parent initiated play is often physical but includes; Imitation, holding, throwing in the air, singing and making sounds, dancing, telling stories, playing with things, laughing together, chatting, tickling, fighting, playing a game, running, jumping, crawling, climbing, massaging. Some of this was obvious to see in the observations, particularly the chatting, tickling, dancing and throwing children in the air. Some caregivers mention singing or chatting to a child particularly before the child was mobile. Some mothers and fathers described play as low priority, even a worthless activity particularly for young babies where they are “not yet aware of things”. Even mothers and grandparents mentioned how others might think you have “gone crazy”, are “mentally disturbed”, “foolish” or “lack anything to do if you are found playing with your child”. Some, however, put this down to a lack of education by members of the community and explained that those who did not play had a lack of love or interest for the child.

**2. The Use of Materials in play.** Parents mentioned homemade toys; “*Ah*, *let me say how I encourage my child* …, *I do make a see-saw…and push him/her” (FGD Malombe Fathers)*. Some parents felt it was a barrier to not be able to buy toys but mainly the barriers come from not having the time and space in the day to play. Caregivers often link play, feeding and bathing. This is a time in the day where play and communication activities are possible. Observations showed that mothers and grandmothers were using a number of materials for the child to play including; balls, sticks, pots and pans, mud and clay to make objects, dolls and cars. For example, one observation in the urban area demonstrates the use of materials for pretend play; “*The mother laughs and goes inside the house*, *she comes back outside carrying a doll*, *a spoon and a small plastic cup*. *At this R*, *she smiles and grabs the doll and cup from her mother dropping the spoon to the ground*. *She picks the spoon up and walks to where she was sitting at first*. *She sits down and begins to play-feed the doll*” (Obs Chilomoni).

**3. Maternal health.** In some instances, both fathers and mothers mentioned that the mother’s health might influence the ability for her to care for the child. In one instance, a focus group of men mentioned the importance of looking after their wives in order to support them looking after their infants. Both mothers and fathers did not commonly voice this. We had no observations where we saw mother’s caring for their own health or well-being.

**4. Responsiveness.** Play and responsiveness may interweave as one and the same. We found this topic difficult to discuss concretely often having an air of innateness with a disjointed approach to gaining data. Many observations clearly showed the responsiveness and a non-verbal understanding and bond between caregivers and children; “*The mother is throwing B in the air and B is smiling* (Obs Chi),” and “*P is shaking her body and it seems she wants to get off her mother’s lap*. *The mother places her on the veranda*”(Obs Mangochi). Language surrounding the idea of responsiveness is unfamiliar and difficult to discuss with clarity. Our focus groups and interviews demonstrate an innate ability by parents to bond and interact with their baby. Responsiveness or interaction with a baby may occur in the form of throwing the baby up in the air, stroking the baby and talking to the baby*”when she is smiling let’s say you are doing this to her it happens that may be you call her mummie mummie” (PC Int Chilomoni) or “Babies play means we should recognise that the child has a spirit” (PC Int Mangochi)*. Having a bond between yourself and the baby is obvious to most parents and responsiveness and early “play” emerge; “*Sometimes your heart can tell you that the child that is crying is yours” (PC FGD Chilomoni) “We have excessive love* … *that is why we play with our children and we start playing with them soon after their birth*, *we must play with them” (PC Int Mangochi)*. Responsiveness may often relate to an understanding of a child’s basic needs as well as specific health needs. “*A lot of problems arise when a parent is not being alert with her child” (IDI Mothers Chilomoni Chibwana)*.

A number of barriers to responsivity were voiced. These included; a lack of ability of mothers to recognise their child’s voice “*You’ll find that a child is crying but the mother is not busy with even to carry it” (FGD Mothers Chilomoni)*. They also included; diverted attention due to poor family planning and closely aged siblings, fear of the father and problems in the home.

**5. Communication.** Physical contact with a child is mentioned as the main form of communication particularly with very small babies*; “You know the child very well and if there is a problem you come to know when you swing the child does not respond positively” (FGD Grandmothers Malombe)*. Parents described singing to teach communication but also physical closeness. *“Communicating with a child*, *when you love the child*, *you stay closer to him/her and vice versa and the child has a lot of worries and feels that the mother does not love him/her*, *if you love the child*, *the child stays closer to you and therefore you do communicate in everything (Mothers IDI Katema)”*.

A number of caregivers described communication as mainly coming from a caregiver to the child rather than from a joint interaction such as a child’s understanding of commands; “*We can talk to a child as we would an adult*, *we can tell it to come so that we carry it or breastfeed it*” (Mothers FGD Sigerege, Chilomoni), “*If a child was getting something and you shout at him he stops getting it…*..*we know that the child can now understand and he is growing (Mother IDI Nankhumba Mangochi)*. In the observations we saw a number of instances of effective communication between caregiver and child, which will support development of language in a child; “*The mother is telling D to look at the cows which are passing by the fence*. *D is looking at the cows and he is saying*, *“bow-wow” (Obs Mangochi)*, *“The mother is asking D that*, *“who is it*?*” as A*, *their sister was passing by*. *The mother is making a sound of a car for D*. *“umh*, *umh*” (Obs Mangochi).

Both caregivers and health workers described feeding and play as important factors in the growth of the child. They did not mention communication as important. Many participants described barriers to communication including an inability to understand signs needed to communicate with a child, a lack of time to talk to the child, being a first time mother, separation due to the need to do chores.

**6. Responsive feeding.** Most mothers felt that they were in tune with what they should do in terms of feeding their infants and children. Observations showed many instances of responsivity but mainly in breast-feeding; “*Mother sits down on the veranda with P already on the breast suckling*. *The mother is playing with P’s face as P suckles*” (Observation Chilomoni). Mothers know the advice to breastfeed their child exclusively until six months. There were many reasons why this was not straightforward. Poverty, hunger and other responsibilities could lead to early feeding of porridge. Caregivers said that they had knowledge regarding complimentary foods but they often cited poverty as a reason that it was difficult to provide nutrient rich foods.

### The Mesosystem

Table 5 demonstrates the barriers and enablers to care, responsiveness, play and stimulation at the level of the family, community and sociocultural situation and highlights ways that agencies could considered when implementation a future package.

**Table 5:** Barriers and enablers to care, responsiveness, play and stimulation at the level of the community and society (Mesosystem and Exosystem).

	Barriers(quotes) and Facilitators (quotes)	Derivation of data (FGD, IDI, Obs)	Implications for intervention development
	**The Mesosystem**		
**1.Safety and protection of children**	“*Where there is water …*.*you need not to take them out of your sight*. *They really like to eat mud… if you don’t see such things the child suffers from different diseases…*. *They can even eat dog poop if the parent is far* .. *a child is a child*.*”*(FGD mothers urban). “*Yah*, *there are problems especially during this coming rainy season*, *the child falls into water…*..*if is not well taken care of” (FGD mothers rural)* “*I would obviously supervise these kids if I had enough time in my schedule…*..*Because it happens sometimes that you are with your friends and you are not busy with what the child is doing*. *Then you will realize that a child has fallen and has hurt himself” (IDI Mother urban)*	FGD, IDI, Obs	Need for safe places for children to play–linkage to community based childcare centres. Support for busy mothers with many responsibilities.
**2.Who cares for the baby**	“*when the child is crying we men don’t know why but mother knows*” (FGD Fathers Mangochi) “*He plays with her nicely and if I am cooking he takes the child and plays …until I finish cooking and the child does not cry…until I finish all chores*” (*IDI Mother Rural*). *“Most of the times like in this area* ….*get a small child and leave him/her with the baby…because they have gone to the field or to do some things” (Mothers FGD Rural)*	FGD, IDI, Obs	Supporting mothers already busy. Others in family need to support mother in caring for the baby Siblings need supervision in looking after children
**3.Fathers**	“*Fathers of today don’t play with children because they are always on the move*. *They are always at the lake and when they come back they go straight to sleep*” (*Grannies FGD Rural)*. “*So some are afraid to care for the child—that is why even if the child has defecated*, *they will stay minutes without cleaning him waiting for the mother to come from the stream and when she is back the husband will… start shouting at the wife to clean the child and that is also one type of cruelty*” (*FGD Fathers Rural*). “*The father is the head of the family and is his responsibility to look after the child and monitor his wife on how she is taking care for a child*” (*FGD Fathers Rural*). *“It happens that* … you have married and maybe you have….*step children*, … *the majority of men don’t care because they are not his so he cannot care just*..*the way he can* .. *for his own child”* (*FGD Fathers Rural*)	FGD, IDI, Obs	Provision of advice on parenting and childcare more specifically for fathers.
	**The Exosystem**		
**1.Responsibilities for mothers**	“*It becomes difficult when you just go to work in the morning while the child is still sleeping without knowing how he is that day maybe the child got sick during the night*. *So when you reach here you find that when your friends are calling you that the child here is not ok that’s when the sickness has become more serious already*. *Because you are not being alert with your child”*. *(FGD Mothers Urban)* *“She is going back into the kitchen and she is setting the fire ready to cook lunch*. *…*.*is crying for the mother and the mother is just going without paying attention” (Rural observation)*. “*The mother is carrying a knife in her hands and container with water in the other*. *She puts the vegetables in the water*, *she sits and begins to cut the vegetables together with some tomatoes*. *R begins to cry and the mother picks her up and put her on the back*. *The mother is cooking while R is on her back*” (Rural observation)	FGD, IDI, Obs	Supporting mothers with their responsibilities with childcare (community base childcare centres) or with microfinance or other cash or social welfare programmes.
**2.Food security and money**	“*In the evening if there is food before 4 o’clock I give them but if there isn’t any food that’s all*.. *that’s it”* *(Mothers FGD Rural)* *“this is what they are fed because we are poor*. *We are knowledgeable but we cannot manage to buy that”* (Father FGD, Rural). *“When you are late from the farm and when you want to make porridge for the child*, *you prepare it when is late and sometimes you eat together with the child during lunch time yet the child stays the whole morning without being fed … ” (Grannies FGD Rural)* *“In this community there is lack of care due to the fact that the child is fed at wrong time due to the fact that we are busy looking for food for the child to eat*..*”* *(Grannies FGD Rural)*.	FGD, IDI, Obs	Microfinance or other cash or social welfare programmes.

Barriers to play at community level were multiple and often related to the interaction between parent, family and the wider socioeconomic situation. Barriers mentioned included; parental illness, separation for work (including housework) and the prioritisation of gaining food or money. This also included others opinions about play as time-wasting activity, mother’s low mood after fighting with the father, safety concerns about area of play or items to play with, interruptions due to the other needs of a child. Caregivers often mooted financial problems as a reason why this was more difficult.

#### 1. Safety and protection of children

Many parents commented on the safety and protection of their children. A lack of supervision is a major concern to many carers. Many parents explain how once their child was mobile, there are many barriers to safety. Our observations demonstrated the parental concern over dirty; “*V is taking something on her hands and the mother is removing it from her*” (Observation Mangochi) and unsafe; “*The mother is again singing for B but she is busy removing another stick from the grass fence and starts hitting it on her mother’s head and the mother is removing it from her*” (Obs Mangochi) environments; “*They can get torn in the flesh with thorns… with glass*” as well as exposure to fires; “*if he/she crawls and if the fire is near……*.*you must keep him/her away from that” (FGD Malombe Mangochi Mothers)*. Parents describe a pressing need for good supervision particularly in urban areas.

Some mothers feel that their circumstances force them into situations of not being able to supervise their children. Some fathers said they could support mothers in a supervisory role*; “That is also another problem that a child faces when crawling but as the mother is doing other things at home*, *the father should monitor how a child is playing …*..*as the father you have to monitor that and at the same time*, *you feel happy that that your child is walking but you make sure that is not playing with broken bottles” (FGD Fathers Malombe*, *Mangochi)*

#### 2. Who cares for the baby–roles of others

The responsibility for care is primarily with the mother particularly for infants. Mothers “*know the child best*” and may have time to “play” but often do not as they have other work to do. Mothers told us that care for the child is therefore affected by the time she has available in her day.

Grannies were both reported and seen to be helpful; *“They are interested in the grandchild and they put it on their lap and even carry it when they are going somewhere” (FGD Mothers Rural)*, *“The grandmother is going to the kitchen with S and they are playing together” (Observation Chilomoni)*. There was sometimes a lack of ownership by grandmothers and a sense that it is mother’s duty to look after the child and that the grandmother has completed her duty already with her own children. Some mothers and grandmothers mentioned nannies, particularly in urban areas, but some of those interviewed were concerned that nannies did not feed children well. We did not see nannies in our observations but we did see other caregivers including uncles, aunts and grandparents.

Siblings are prominent in all our discussions; *“But sometimes the child’s sibling helps it to play because there may be 2 or 3 children at home” (IDI PC Chilomoni)*. Some caregivers explained that older siblings could be a necessary source of childcare for smaller children in the family; *“You give it to other siblings to play with when you are busy working and tell the siblings to take care of it when they are playing outside while you are working*. *(PC IDI Malombe*, *Mangochi)*.

Many respondents describe a communitarian approach to childcare where a good member of society support other’s children**: *“****When I see a child crying*, *I pick it up and take it to my fellow mother saying ‘‘ did you not see that the child is crying or has fallen”*? *When a child gets hurt you don’t have to wait for its mother to take care of it*, *you are responsible for that too”*. *(FGD Chilomoni)*

#### 3. Choice and investment for fathers

Generally, there was a lack of participation of the father implied by mothers. Often the father is away attempting to support the family financially (including in South Africa). Some fathers may not feel confident with child caring; “*he says he’s afraid he’ll smell of urine*” (*FGD Mothers Chilomoni*). Mothers and grandmothers often described a father’s role as limited in terms of their care for young children. For example, fathers might mind the child for a limited period while mothers do house work. Some fathers were very confident with their care and wanted to play an active role more so in providing food and money and having their children see them as head of the household. Some grandmothers were suspicious of fathers; *“*They don’t play with them. They go and have sex with others after they have seen that she has given birth.” Fathers were available in some observations and were mildly involved with interacting with the child but for very short periods.

In some interviews, fathers described how they often play a managerial role in the family, being the ones who tell mothers how best and what to do with their children. Some mothers said that fathers had “cruel intentions”. Men often talked about a “choice” in looking after or playing with a child and sometimes this was dependent on whether it was a stepchild or orphan or their own child.

### The Exosystem

#### 1. Mother’s responsibilities

We found that all our transcripts had descriptions streaming through them of the multiple responsibilities undertaken by women. Mothers are constantly busy with chores such as preparing food, knitting or making fires. These responsibilities often mean that mother’s attention is away from the child. In all observations, mothers were busy with chores whilst supporting and caring for their children and seemed to have little time to “play” with their children. Some mothers talked poignantly about these difficulties to the point that their child becomes sick. A number of mothers were seen “backing” their child (putting them on their back) to enable them to get on with chores; “*In the kitchen the mother is still backing V and is putting V down to facilitate the processing of making nsima*” (also see Table 5) or directing their child to help with chores, even from 20 months of age, for example, “*The grandmother is calling J to go to the kitchen and help her cooking*, *the grandmother is telling J to go and get a knife from the neighbour but J is just listening to the playing songs*” (Obs Mangochi). A mother’s physical and mental health is also very important in supporting the care of her children. Much of this discussion came from father’s FGDs and solidified the importance of nutritional support not just for infants but also for mothers looking after them.

#### 2. Food security and money

All those who were interviewed described food security and resources as placing an enormous barrier on their ability to care for the child. This includes not having the resources to buy enough food as well as complementary foodstuffs. Fathers explained that they may know what they need to provide the children but they did not have the resources to do so.

Mothers reported how the necessity to farm and work to provide food affects their abilities to care for children. *“Problems of not having food at home*, *we want to go and search for food for the children to eat*. *There is a problem because you wake up in the morning and you don’t cook for the child because you don’t have flour for porridge or any money*. *You then run off in search for money in order to feed the children*” (PC FGD urban). Mothers were not always able to be present for observations as they were working in the field or were busy gathering vegetables; “*The mother is telling her brother to help her picking V as she wants to pick some mangoes*. *She is coming back to the house with mangoes in her hands and putting V down*” (Observation Mangochi).

## Discussion

The information emerging from this study provides us with an understanding barriers and facilitators relevant to the promotion of play, responsiveness, communication and feeding of the young child in Malawi. This is particularly timely considering the recent release of the nurturing care framework[13]. This study has provided the groundwork for understanding the issues, which need to be addressed if training packages supporting the care of young children in Malawi are to be implemented. Our study also provides a good example of the early formative research that should be undertaken prior to creating specific programmes which target the care, stimulation and feeding of children from 0–2 years [6, 9].

It is clear that barriers and facilitators exist at all levels of the ecosystem and that without addressing support need at all levels, programmes will be ineffective. Our study is likely to be relevant to many settings in Sub-Saharan Africa, particularly those in resource-limited settings where international agencies are particularly targeting the use of integrated programmes to support early child development.

Certain themes came up regularly within our study. These included; the importance of health, purpose of play–a child who plays will be well, knowledge of infant hearing and vision, parent initiated play, the use of materials in play, maternal well-being and mental health, responsiveness, communication and talking to your child early on, responsive feeding, child protection, who else looks after the baby, father’s roles, maternal responsibilities and food security, resources and money (Table 2).

Caregivers may want to invest time in playing, communicating and interacting with their children however many describe how they have so many competing demands within the household, at work and in the fields primarily to provide resources for the family are of course, much higher up the agenda. Recent studies in Malawi have even demonstrated how much more accepting children can be of food if the mother takes a more responsive approach to feeding [39]. Our data demonstrates how some carers who invested time in stopping to play, interact, and stimulate their children can be seen as seen as “mad” or lazy by others in the community. Research in other locations has similarly demonstrated how mothers and other caregivers may have the knowledge relating to the provision of the right care, but cultural and societal barriers as well as lack of resources prevent them from doing what they know might be best [40]. In Malawian society, women also have many more of these responsibilities than men [41]. It is likely that interventions, which could support mothers in freeing up her time, will succeed more in the longer term. For example, efforts to provide childcare in places where mothers may be working providing safe play spaces and basic care and nutrition in farmer’s cooperatives in Zimbabwe[42]. It is unrealistic to think that interventions will be effective without considering these needs.

Most mothers and carers are clearly responsive to the needs of their children and carers see that health and nutrition are the essence to a child being well. In Malawi, the community promote care of the child as a community undertaking. Parents do not so commonly conduct carer-initiated play and often siblings and friends will play and care for infants and toddlers—particularly when they are old enough to mobilise. This is similar in many African countries [16, 23]. Although evidence on a wider scale does demonstrate that early promotion of stimulation and responsiveness programmes can be effective, it is necessary that the implementation of these programmes is undertaken in an effective and sustainable way [9]. We would advocate that programmes, which simply provide advice to mothers to encourage more play and stimulation, are futile if they do not consider better ways to include siblings and other family members in supporting the care of young children. Furthermore, they need to provide support for already overburdened mothers. This may be possible in Malawi if linked to centre based programmes [43] and is certainly being promoted more within the recent Nurturing Care Framework guidance[13]. Our study benefited from conducting interviews and focus groups with both fathers and grandfathers (as well as grandmothers). Many fathers describe childcare as a mother’s role but then provide “expert opinions” on how it should be done. It is clear that without engaging fathers more in the development of their children, we will create programmes, which do not fully support the family. Recent research from South Africa promotes the idea that men’s needs also should be listened to and engaged with through community programmes[44]. Interventions from Vietnam show how programmes that have specific interventions for fathers can be helpful [45]. There is little research from African settings to provide evidence as to whether something similar could work however there is now advice and support for programmatic work to support fathers within the Nurturing Care Framework[46]. Further research in this area could be very beneficial.

### Strengths and limitations

This study was extensive and included a wide range of participants purposively sampled from a number of different settings of Malawi. We utilised four different methods of data collection to enable us to triangulate data from a variety of sources and to validate themes that emerged from the data. This included respondent feedback to enable us to ensure our results were meaningful.

We had no participants from the Central and Northern parts of Malawi take part in the research due to limitations on time and transportation costs. This may have influenced the data, which emerged within our study, and we may have had different views from those who belonged to different tribes and groups within these areas. Our research team is diverse and includes both individuals native to a number of different regions of Malawi as well as those from the UK. Some team members are medically trained and therefore may have demonstrated some bias in their views on the current topic. Finally, we had few participants who refused to take part in the study but we must be aware that the views of those taking part in this research were only from a limited number of individuals and those who had more problems with caregiving with their children might not have decided to take part. Although we sampled randomly within the villages, mothers who had had difficulties with a child who had become malnourished or neglected, those who had mental health problems and younger mothers may have been less likely to speak out during the FGDs and IDIs and we may not have gained their views. Further studies to identify these mothers and gain their views in particular would be helpful to provide further information for mothers who are struggling more in caring for their infants.

## Conclusions

In implementing a programme to provide advice on developmental stimulation, play, early communication, barriers relating to a caregivers understanding of play, early communication and responsiveness are important to address. Agencies and organisations who are considering incorporating programmes that promote one to one developmental stimulation and care of infants should consider how the multiple responsibilities of caregivers are addressed. Furthermore, agencies may want to consider how these programmes can be adapted to embrace and utilise the multiple caregivers who look after a child in African settings such as Malawi.

## Supporting information

S1 Text Topic guides for FGDs and IDIs CCD Malawi. (DOCX)

S2 Text Guide for structured observations. (DOCX)

S1 Table Supplementary Table 1 ISSM_COREQ_Checklist. (PDF)
