# Supplementary material for: The care, stimulation and nutrition of children from 0-2 in Malawi—Perspectives from caregivers; "Who’s holding the baby?"
Source: PLoS One. 2018 Jun 27;13(6):e0199757. doi: 10.1371/journal.pone.0199757 (PMC6021079; doi:10.1371/journal.pone.0199757)
Supplement: S1 Text — (DOCX) [file pone.0199757.s001.docx]

## Supplementary File 1: TOPIC GUIDE FOR CARER’S FGDs and IDIs

Focus group/interview guides will be adapted and refined during the study according to themes/findings emerging from initial interview data, so are a **basic provisional guide** at present.

| **Topic:** Families/carers experience of feeding and stimulating and playing with children under two years | |
| --- | --- |
| **Material Needed:**  • Participant information sheets  • Flipchart easel + paper  • Pens and papers for the participants  • Markers  • Topic guide  • Dictaphone, notebook and pens for taking notes.  • Pilot tools for training programme e.g. pictorial manuals  **Time limit:** 1 hour to 1h 30 min  **Population group:** Family members/carers (>16yrs) of child under age 2 years in the community  **Number of participants:** 6 to 8 for FGDs, 1 person individually for interviews | |
| **Topic areas** | **Comments** |
| **Objectives of the focus group** | **• Explain briefly the objectives of the discussions**  **• The objectives of the Focus Group discussion are:**  To explore the experience of carers in looking after children in the community with regards to play and communication and feeding of under two year olds. To elicit what information parents and carers have about engaging in play and stimulation and feeding of children under the age of two and to elicit where this information comes from. |
| **Participant’s consent** | **Go through information sheet and take general consent for focus group**  Remind that FG will take up to 1 ½ hrs, participation is voluntary, not taking part will not affect them in any way, they are free to leave at any point, any information they give is confidential within the group, and the discussion will be recorded on Dictaphone but names will not be included and participants will not be identifiable in any reports. |
| **Introductions and ground rules** | **Use an ice breaker to relax the group/individual and get everyone to introduce themselves**. Important to recognise that Focus Groups can be stressful for participants, especially if they do not know each other at all.  **Need to establish some ground rules for the group/individual** (these will be written up on flipchart and participants encouraged to add any extra ones they feel are important):   - Encourage participants to speak one at a time and avoid interrupting each other – this will help everybody hear what is being said and for us to be able to listen back more easily to the recording. - It is important for us to hear everyone’s ideas and opinions, and that everybody’s contribution is equally represented and respected. - There are no right or wrong answers to questions – just ideas, experiences and opinions, which are all valuable. - It is important for us to hear all sides of an issue – both the positive and the negative. - Regarding disclosure – explain that you can’t guarantee absolute confidentiality in a group but suggest that people could talk about “people they know” rather than their own personal experience. Reiterate the importance of confidentiality – what is shared in the room stays in the room, except in the case where child protection concerns are raised – these may need to be shared. |
| **Discussion themes (see detailed guide below)**  Feeding around birth  Difficulties feeding around birth  Feeding later  Difficulties feeding the slightly older infant  Weaning foods  The small baby and feeding  The small baby and weaning  Looking at your child  Talking to your child  Time spent in play  Toys/materials  Difficulties doing play  Who plays | **Potential Activities**  Brainstorm the main things that they know in each area.  Brainstorm the problems that they face with each area.  Write/draw them onto separate pieces of paper (facilitator can help with this) then organise into priority list of the problems they think are the MOST important to get help with.  Brainstorm the main things that parents would like to know.  Hand out pictures of children feeding and get them to talk about the picture (pictures from Care for Development package).  Also pictures of children and carers communicating and get them to brainstorm.  Similar – pictures of children and carers playing |
| **Wrap up & Debrief**  Referral to external services    Recording additional observations | Thank the participant/s and summarise the activities and how the Focus Group/interview will help inform the study. Don’t try to summarise the content of the discussions at this stage.  Ask the participants if there is any information they need and let them know that you will be available after the Focus Group/interview to answer any questions.  Assess if any participants need to receive specialised support/advice and/or need referring to specialist services.  Collect Dictaphone, gather all notes from the note taker and from the person doing the observations. Note down any additional observations directly after the Focus Group. Debrief with focus group facilitator – What went well/badly? Any extra observations? Anything to change for next time? |

Discussion themes:

FEEDING

Feeding around birth

Difficulties feeding around birth

Feeding later

Difficulties feeding the slightly older infant

Weaning foods

The small baby and feeding

The small baby and weaning

Sitting and eating – nsima and porridge... who helps..

Checking child has had enough?

How do you know child has had enough... food/milk/porridge

COMMUNICATION...INFANTS/BABIES/TODDLERs

Looking at your child? When do you? How often?

Hearing..when can a baby hear? From birth? Can they hear?

Seeing – when can a baby see? From birth? Can they see?

Talking to your child/infant/BABY from a few months of age....??timeline???

Talking to your child... how? Harshly...sensitively...

Talking whilst feeding?

Songs – how often..what

Rhymes? Speaking WITH your child... mother/father to child

Responsive..How do you know that the baby responds knows what you are doing...

Listening to your child?

Get down to their level..

Child talking to you..

What age? When do you start talking to your child?

When does your baby talk to you? From what age? Do they just communicate by crying??

Do they communicate in other ways before that?

Do you help them talk to you?? How

Pointing

Sharing pictures or objects...that time when they are just learning their first words?? How is that encouraged??

PLAY

Who plays with who (BABIES/TODDLERS).....

Mums and play

Other children and play

Age at play

Babies playing? Do babies play? How?

Time spent in play – CLOCK.. amount of time in the day...when is there time for play with children?

Toys/materials - what do you play with WITH your child?

Do you make things for your child?

Difficulties doing play

Who plays?

How do children and babies learn and develop?

Is play important?

AWARENESS of what your baby or child is doing

Where are they? What do they play with?

Sensitivity to children? Movements, cues, ideas

Responsiveness to children
