# Supplementary material for: The care, stimulation and nutrition of children from 0-2 in Malawi—Perspectives from caregivers; "Who’s holding the baby?"
Source: PLoS One. 2018 Jun 27;13(6):e0199757. doi: 10.1371/journal.pone.0199757 (PMC6021079; doi:10.1371/journal.pone.0199757)
Supplement: S2 Text — (DOCX) [file pone.0199757.s002.docx]

**OBSERVATION SCHEDULE FOR CCD PROJECT**

The precise nature of these situations is difficult to predict, but we anticipate this would likely include observation at different times of day but may be in relation to morning activities, lunch time or afternoon activities.

1. Context:
   1. Time
   2. Description and context
   3. Description of participants
2. Record of key actions of the participants (for 10 minutes, one minute intervals; followed by interpretation and repeated as required).
3. Interpretation of the situation (by the observer) (five minutes following 10 observations). Focus upon activities which provide an interaction with the child – use of *language*, *actions* and *tools (objects, toys, etc.)*

*.*

**ID Number: Mother’s age:**

**AREA: Number of children mother has:**

**AGE: 1YEAR 8 MONTHS OLD Father living in household: Y/N**

**GENDER: Observer:**

**Date:**

**Note about time arriving:**

| **Time** | **Activity Record** | **Language Record** | |
| --- | --- | --- | --- |
|  |  |  | |
|  | **Interpretation:** | | |
|  |  |  | |
|  | **Interpretation:** | | |
|  |  |  | |
|  | **Interpretation:** | | |
|  |  |  | |
|  | **Interpretation:** | | |
|  |  | |  |
|  | **Interpretation:** | | |
|  |  |  | |
|  | **Interpretation:** | | |
|  |  | |  |
|  | **Interpretation:** | | |
|  |  |  | |
|  | **Interpretation:** | | |
|  |  | |  |
|  | **Interpretation:** | | |
